# Examination of Runs of Homozygosity Distribution Patterns and Relevant Candidate Genes of Potential Economic Interest in Russian Goat Breeds Using Whole-Genome Sequencing

**DOI:** 10.3390/genes16060631

**Published:** 2025-05-24

**Authors:** Tatiana E. Deniskova, Arsen V. Dotsev, Olga A. Koshkina, Anastasia D. Solovieva, Nadezhda A. Churbakova, Sergey N. Petrov, Alexey N. Frolov, Stanislav A. Platonov, Alexandra S. Abdelmanova, Maxim A. Vladimirov, Elena A. Gladyr, Igor V. Gusev, Svyatoslav V. Lebedev, Darren K. Griffin, Michael N. Romanov, Natalia A. Zinovieva

**Affiliations:** 1 L.K. Ernst Federal Research Center for Animal Husbandry, Dubrovitsy, Podolsk Municipal District, 142132 Podolsk, Russia; horarka@yandex.ru (T.E.D.); asnd@mail.ru (A.V.D.); olechka1808@list.ru (O.A.K.); anastastasiya93@mail.ru (A.D.S.); nadushik95@mail.ru (N.A.C.); citelekle@gmail.com (S.N.P.); abdelmanova@vij.ru (A.S.A.); maksim-taranov@rambler.ru (M.A.V.); elenagladyr@mail.ru (E.A.G.); igorgusev@mail.ru (I.V.G.); n_zinovieva@mail.ru (N.A.Z.); 2 Federal Research Center for Biological Systems and Agrotechnologies of the Russian Academy of Sciences, 460000 Orenburg, Russia; forleh@mail.ru (A.N.F.); platonstas1994@mail.ru (S.A.P.); lsv74@list.ru (S.V.L.); 3 School of Natural Sciences, University of Kent, Canterbury CT2 7NJ, UK; 4 Animal Genomics and Bioresource Research Unit (AGB Research Unit), Faculty of Science, Kasetsart University, Chatuchak, Bangkok 10900, Thailand; 5 Royal Veterinary College, University of London, London NW1 0TU, UK

**Keywords:** goat (*Capra hircus*), Russian local breeds, whole-genome sequencing (WGS), runs of homozygosity (ROHs), signatures of selection, candidate genes

## Abstract

Background/Objectives: Whole-genome sequencing (WGS) data provide valuable information about the genetic architecture of local livestock but have not yet been applied to Russian native goats, in particular, the Orenburg and Karachay breeds. A preliminary search for selection signatures based on single nucleotide polymorphism (SNP) genotype data in these breeds was not informative. Therefore, in this study, we aimed to address runs of homozygosity (ROHs) patterns and find the respective signatures of selection overlapping candidate genes in Orenburg and Karachay goats using the WGS approach. Methods: Paired-end libraries (150 bp reads) were constructed for each animal. Next-generation sequencing was performed using a NovaSeq 6000 sequencer (Illumina, Inc., San Diego, CA, USA), with ~20X genome coverage. ROHs were identified in sliding windows, and ROH segments shared by at least 50% of the samples were considered as ROH islands. Results: ROH islands were identified on chromosomes CHI3, CHI5, CHI7, CHI12, CHI13, and CHI15 in Karachay goats; and CHI3, CHI11, CHI12, CHI15, and CHI16 in Orenburg goats. Shared ROH islands were found on CHI12 (containing the *PARP4* and *MPHOSPH8* candidate genes) and on CHI15 (harboring *STIM1* and *RRM1*). The Karachay breed had greater ROH length and higher ROH number compared to the Orenburg breed (134.13 Mb and 695 vs. 78.43 Mb and 438, respectively). The genomic inbreeding coefficient (*F*_ROH_) varied from 0.032 in the Orenburg breed to 0.054 in the Karachay breed. Candidate genes associated with reproduction, milk production, immunity-related traits, embryogenesis, growth, and development were identified in ROH islands in the studied breeds. Conclusions: Here, we present the first attempt of elucidating the ROH landscape and signatures of selection in Russian local goat breeds using WGS analysis. Our findings will pave the way for further insights into the genetic mechanisms underlying adaption and economically important traits in native goats.

## 1. Introduction

The utilization of large-scale genomic data to search for selective sweeps and genome-wide associations in the genetic make-up of domestic goats (*Capra hircus* Linnaeus, 1758; CHI) is key to pinpointing changes pertaining to their domestication, breed formation, and recent selection [1]. For instance, based on whole-genome sequencing (WGS) data in Pakistani Teddy goats, Saif et al. [2] found genes in selective sweep regions that were associated with body weight, reproduction, milk production, litter size, wool production, and coat color. In another investigation using WGS data [3], strong selective signatures were detected that harbored novel genes associated with cashmere traits and environmental adaptation. Gudra et al. [4] identified 26 genetic variants associated with somatic cell count in Latvian local goats. GWAS performed in Leizhou goats revealed a significant variant (g.53666634T>C) affecting leg length traits [5]. In dairy goats, Xiong et al. [6] identified genes associated with milk production traits and reproduction traits. Sheriff et al. [7] detected nine strong regions spanning 163 genes that may affect adaptation to arid and semi-arid environments and immune response in Ethiopian indigenous goats. Wang et al. [8] proposed a number of genes as potential candidates for fecundity traits in highly prolific Jining gray goats.

Searching for runs of homozygosity (ROHs) is a widespread genomic and bioinformatic approach used to detect selection signatures in livestock species [9,10,11,12,13]. Many reports have involved the identification of candidate genes overlapping with ROH islands and related to economically important and adaption traits in farm animals [14,15,16,17,18]. For example, using whole-genome resequencing data, Li et al. [19] and Zhao et al. [20] applied this approach to find candidate genes that are responsible for reproduction traits, hair follicle development, fat tail formation, growth, and feed intake in Chinese Hu sheep. Analyzing the whole genomes of 100 American minks, Davoudi et al. [21] detected 63 ROHs embracing genes associated with fur quality, body size, and reproduction. Ayalew et al. [22] analyzed whole-genome sequences of African zebu cattle to address genetic variants associated with heat tolerance and high immune resistance. In the case of domestic goats, Sun et al. [23] identified the *IL2*, *IL7*, and *KIT* genes that overlapped with ROH islands and were involved in immune processes in Jianchang Black goats. Signer-Hasler et al. [1] performed an analysis of 226 caprine genomes from 11 populations and identified ROH islands possessing 2 candidate causative genetic variants that might be related to domestication.

**Figure 1:**
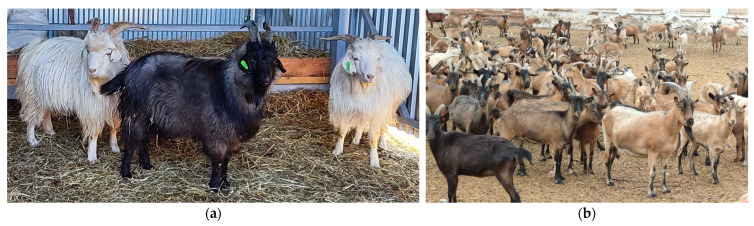
The representative studied goat breeds of Russian origin: (**a**) a family of Orenburg goats (a black male and two white females); and (**b**) a herd of Karachay goats.

The Orenburg goat breed (Figure 1a) is an endemic and landmark breed that produces soft cashmere fibers [24,25,26]. This breed, created in the Orenburg province near the Urals Mountain, is adapted to harsh continental climate conditions and not able to manifest high-quality down performance in other regions. The breed gained world fame when a down-knitted shawl made from Orenburg goat down won golden medals at the World Fair [27]. In addition, among the variety of local goat breeds, the Orenburg breed is one of the most prolific. The fertility of goats of this breed averages 140 kids per 100 goats. About 52.6% of females give birth to twins, and less frequently to triplets (about 2%) [28].

The reducing demand for down-knitted pieces has led to a dramatic decrease in the population size of this breed. In the crisis state of the down knitting industry, Orenburg goats are also used to obtain, in addition to down, other products, including meat [29,30]. Indeed, Orenburg goats can be raised under intensive breeding conditions, demonstrating excellent meat quality [29].

The surveillance of modern remaining populations and genetic monitoring using microsatellite markers [31] and single nucleotide polymorphism (SNP) arrays [32] have revealed that the original genomic components remain in the Orenburg breed. Currently, the initial steps to conserve the gene pool of this endemic breed are fortunately taking place by implementing its gamete preservation [32].

The Karachay breed (less often *Karachai* [33,34,35,36], sometimes *Karachaevskaya* [33,37] and *Karachaev* [37,38,39], the latter name being not entirely correct) originates from North Caucasus regions and is characterized by stamina, unpretentiousness, and high resilience to the local conditions [40] (Figure 1b). The birth rate of Karachay goats is within the range of 1.19–1.26 [34,41]. This is a dual-purpose breed (i.e., used for meat and milk), and the goats may be raised under conditions of ecologically friendly maintenance on mountain and foothill pastures [35]. Importantly, a few genomic studies were previously conducted to address their production traits. In particular, a GWAS revealed several genes that were significantly associated with the dry matter content and fatty acids in the milk of Karachay goats [36]. In addition, Selionova et al. [35] reported that a GG genotype at a single SNP locus, rs268269710, was associated with a heavier live weight and a greater carcass yield in young Karachay goats. Despite a complex of beneficial traits, indigenous Karachay goats are not as highly productive as commercial breeds [42]. Because of that, this breed is used as a maternal breed to develop the first Russian meat goat breed by crossing Karachay dams with Kalahari Red sires [39].

In our previous studies [38,43], we addressed the genetic diversity and population structure of the Orenburg and Karachay goat breeds based on SNP genotype data. However, the detection of selection signatures in the genomes of Orenburg and Karachay goats underlying adaptability and other economically important traits is relevant and will be instrumental for their further conservation, sustainable maintenance, and performance improvement. In this respect, the aim of the present study was to identify the breed-specific ROH landscape, potential selective sweeps, and candidate genes located within ROH islands in the Orenburg and Karachay goat breeds based on WGS analysis.

## 2. Materials and Methods

### 2.1. Animal Sampling

The samples utilized in this study were represented by tissue specimens of goats from the Orenburg (*n* = 19) and Karachay (*n* = 20) breeds, two Russian local breeds that originated in different environments under different selection pressures and characterized by different production performances. The sample sizes of the two breeds assigned to whole-genome sequencing were limited by the funding policy annual restrictions, while being compensated by the high depth of sequencing coverage (see Section 2.2). For ethical animal handling and sampling, see the Institutional Review Board Statement section. Prior to collecting samples, the following procedures were performed. The 1.7 mL Eppendorf tubes were numbered and filled in with 1 mL of 95% ethyl alcohol. To collect auricular samples from the goats, tagging forceps for livestock were used. The skin sampling sites were aseptically cleaned to avoid contamination. All procedures were performed by trained personnel wearing sterile medical gloves. The size of the auricle samples was not less than 0.5 by 0.5 cm. After collection, each ear biopsy was immediately placed in an Eppendorf tube, with ethyl alcohol completely covering a sample. The tubes were closed tightly and transported to the Center for Collective Use of Research Equipment “Bioresources and Bioengineering of Agricultural Animals” at the L.K. Ernst Federal Research Center for Animal Husbandry (LKEFRCAH).

For this study, we selected only typical, healthy, unrelated individuals that met the breed standards. The kinship and breed assignment of the sampled goats were preliminarily checked by microsatellite analysis. Sample collection of the Karachay goats (females) was performed on one flock at the Ladozhsky breeding farm, an LKEFRCAH branch situated in Krasnodar Krai, South Russia (45.304996, 39.891568), on 24 March 2020. The local climate is dry subtropical, transitional from moderate continental with long hot summers and warm winters (the average annual temperature is about +13.3 °C). The average age of the goats varied from 1.5 to 2.2 years. Their fiber color was brown and red-brown. Sampling from the Orenburg breed was performed on three flocks in three locations in Orenburg Oblast (formerly the Orenburg province) (51.135854, 57.945351, 51.289560, 58.178482, and 51.355471, 56). The climate is continental, with warm summers (along with frequent droughts) and cold winters. Ten Orenburg samples were collected at the Guberlinsky nucleus farm on 16 March 2017, and included three sires and four dams aged four years and three young females aged one year. Nine females (aged 2–3 years) were collected from two small holders’ farms on 19 March 2024. The fiber color of all studied goats was gray.

**Figure 2:**
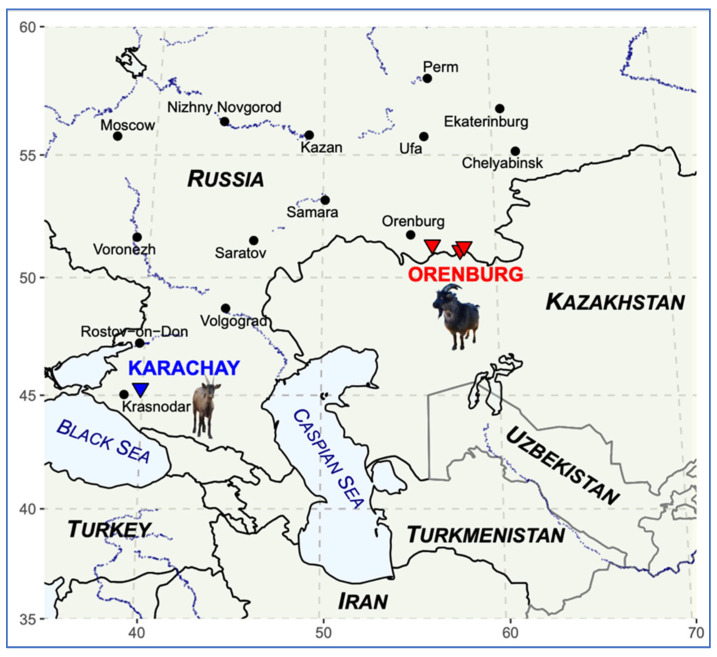
The map with the corresponding sampling sites of goats for the Orenburg (▼) and Karachay (▼) breeds.

Figure 2 shows the sampling locations for the Orenburg (marked in red color) and Karachay breeds (marked in blue color), along with silhouettes of the representatives of the studied breeds. The map of the sampling sites (Figure 2) was created using R packages maps (Version 3.3.0) [44] and ggplot2 (Version 3.5.2) [45].

### 2.2. DNA Extraction and Whole-Genome Sequencing

Genomic DNA was isolated from tissue samples using the DNA Extran 2 kit (Syntol, Moscow, Russia). In order to assess the quantity and quality of the double-stranded DNA, its concentration was determined using a Qubit™ fluorometer (Invitrogen/Life Technologies, Waltham, MA, USA), while the ratio of absorption at 260 and 280 nm (OD 260/280) was measured using a NanoDrop 8000 device (Thermo Fisher Scientific Inc., Waltham, MA, USA). The minimum amount of DNA used to prepare sequencing libraries was 3 μg; therefore, the threshold DNA concentration was 30 ng/μL in at least 100 μL volume. When the concentration was lower, the DNA was subject to re-isolation in order to enhance the quantity of the initial material. In addition, to check the integrity of the DNA, the electrophoresis was run in 1% agarose gels.

To make the sequencing libraries, TruSeq DNA Nano Library Prep kits (Illumina, Inc., San Diego, CA, USA) and the Accel-NGS^®^ 2S Plus DNA Library Kit (Integrated DNA Technologies, Inc., Coralville, IA, USA) for Illumina^®^ Platforms (Swift Biosciences, Inc., Ann Arbor, MI, USA) were utilized. Paired-end libraries with a fragment size of 150 bp were constructed for each goat. Next-generation sequencing was performed using a NovaSeq 6000 sequencer (Illumina, Inc., USA). Genome coverage was 19×–20×. Alignment to the reference goat genome assembly ARS1.2 (https://www.ncbi.nlm.nih.gov/datasets/genome/GCF_001704415.2/, accessed on 25 April 2025, https://ftp.ensembl.org/pub/release-113/fasta/capra_hircus/dna/, accessed on 25 April 2025) was executed using bwa-mem2 tools (Version 2.2.1) [46] and SAMtools (Version 1.21) [47].

### 2.3. Data Processing and in Silico Analyses

A total of 20,554,866 SNPs were used for ROH identification. Prior to further constructing Neighbor-Net graphs and performing principal component analysis (PCA), the SNPs were pruned using their linkage disequilibrium criterion. After pruning, a total of 4,588,309 SNPs were left for the subsequent analyses. Pairwise identity-by-state (IBS) distances were computed using PLINK v1.9 [48] (with the software setting --distance 1-ibs). The Neighbor-Net graphs based on the matrix of IBS distances were visualized using SplitsTree 4.14.5 software [49]. PCA was performed using PLINK v1.9 [48] and visualized using the R package ggplot2 [45].

The ROH regions were identified using sliding windows in the R package detectRUNS [50] using the following program settings: runs <- slidingRUNS.run(‘outdata_roh.ped’, ‘outdata_roh.map’, windowSize = 50, threshold = 0.05, minSNP = 50, ROHet = FALSE, maxOppWindow = 1, maxMissWindow = 5, maxGap = 100,000, minLengthBps = 100,000, minDensity = 1/10,000. Herewith, the following parameters were used to identify the ROHs:

(1) windowSize is the size of the sliding window (i.e., number of SNP loci; default = 15);
(2) threshold is the threshold of overlapping windows of the same state (homozygous/heterozygous) to call an SNP in a RUN (default = 0.05);
(3) minSNP is the minimum number of SNPs in a RUN (default = 3);
(4) ROHet is a heterozygosity/homozygosity parameter for whether runs of heterozygosity (ROHet) or homozygosity (ROHom) are detected (default = FALSE);
(5) maxOppWindow is the maximum number of homozygous/heterozygous SNPs in the sliding window (default = 1);
(6) maxMissWindow is the maximum number of missing SNPs in the sliding window (default = 1);
(7) maxGap is the maximum distance between consecutive SNPs to be still considered a potential run (default = 10^6^ bp);
(8) minLengthBps is the minimum length of a run in bp (defaults to 1000 bp = 1 Kb);
(9) minDensity is the minimum number of SNPs per Kb (defaults to 0.1 = 1 SNP every 10 Kb);
(10) maxOppRun is the maximum number of opposite genotype SNPs in the run (optional);
(11) maxMissRun is the maximum number of missing SNPs in the run (optional).

ROH segments shared by at least 50% of the samples were considered as ROH islands.

We calculated ROHs for each goat and then categorized ROHs according to the following four length bins (classes): 0.1–0.2 Mb, 0.2–0.4 Mb, 0.4–0.8 Mb, and >0.8 Mb. For each breed and length class, the total number of detected ROHs was computed for each individual. To determine the mean sum of ROHs, the total ROH length for each goat in the populations was calculated, and the results were averaged by breed group.

The genomic inbreeding coefficient based on ROHs (*F*_ROH_) was computed as the sum of the length of all ROHs per goat proportioned to the total autosomal SNP coverage.

## 3. Results

### 3.1. Between-Breed Genetic Differentiation

**Figure 3:**
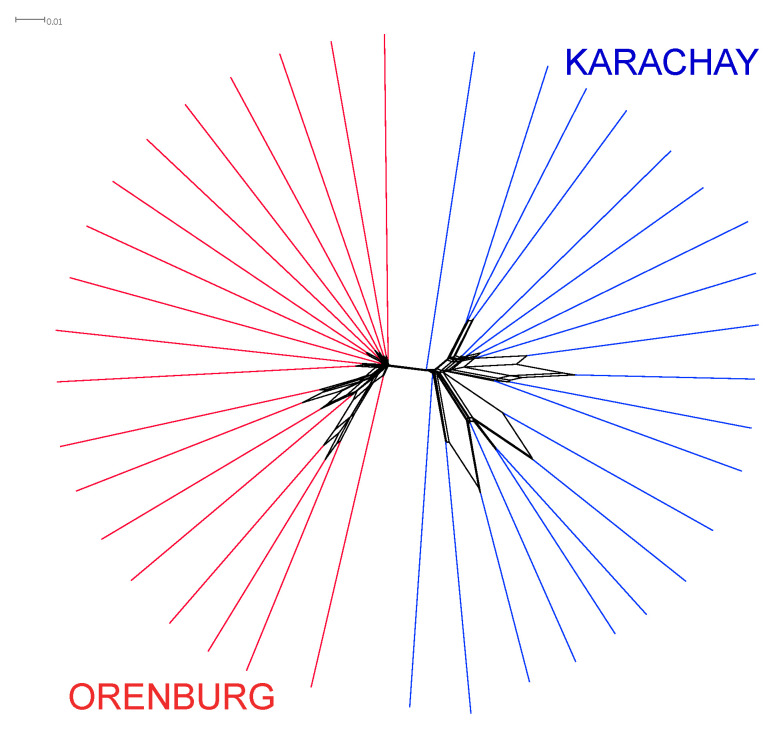
Individual Neighbor-Net graph using IBS distances plotted for the Orenburg and Karachay breeds based on the WGS data analysis.

As an initial step, we investigated a pattern of genetic relations and differentiation between the two studied goat breeds. Figure 3 shows the Neighbor-Net plot for the Orenburg and Karachay breeds. The breeds were clearly differentiated by forming their own clusters.

**Figure 4:**
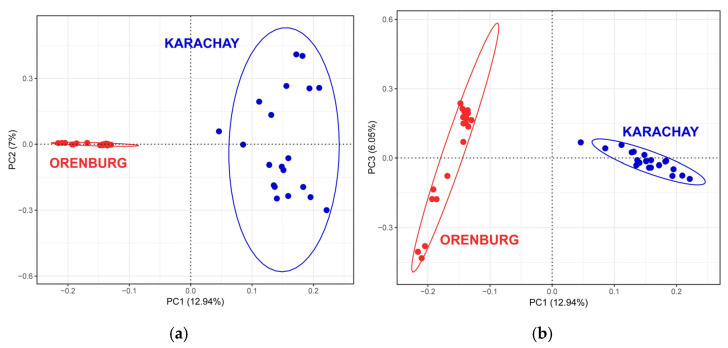
Principal component analysis performed for Orenburg and Karachay goats based on the WGS data analysis: (**a**) for the first two principal components (PC1 and PC2); and (**b**) for the first and third principal components (PC1 and PC3).

PCA revealed that PC1 apparently differentiated the Orenburg goats from the Karachay breed (Figure 4), and this distinct differentiation pattern for the two breeds was consistent with the Neighbor-Net analysis plot (Figure 3).

### 3.2. Runs of Homozogosity Patterns

As a result of the subsequent in silico analysis, we found out that the Karachay breed had a greater ROH length and higher ROH number in comparison with the Orenburg breed (Table 1). Regarding the individual values of these indicators, the maximum values of the ROH number and ROH length varied from 540 to 2075 and from 102.96 to 404.4 Kb in the Orenburg and Karachay breeds, respectively.

The values of *F*_ROH_ varied from 0.032 in the Orenburg breed to 0.054 in the Karachay breed. The maximum value of *F*_ROH_ detected in the Karachay breed was four times higher than that computed for the Orenburg breed (0.16 vs. 0.04, respectively).

**Figure 5:**
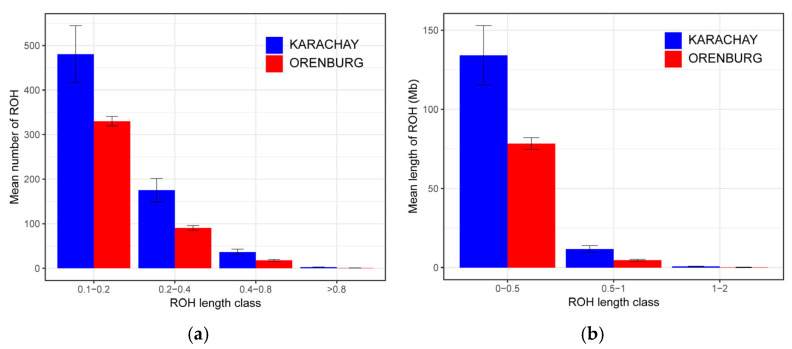
Distribution of runs of homozygosity (ROH) according to length classes in Orenburg and Karachay goats based on the WGS data analysis: (**a**) mean number of ROHs per breed; and (**b**) mean length of ROHs per breed.

When dividing the ROHs into length classes (Figure 5), only short ROHs were identified. The ROHs accounted for class 0.1–0.2 Mb were the most frequent in the studied breeds (Figure 5a). The mean number of ROHs for class 0.1–0.2 Mb was 329.95 ± 10.79 and 480.85 ± 63.46 in the Orenburg and Karachay breeds, respectively. In class 0.2–0.4 Mb, the mean number of ROHs was 90.47 ± 5.42 for the Orenburg breed and 175.15 ± 26.23 for the Karachay breed. The ROHs from class 0.4–0.8 Mb were less frequent and varied from 17.79 ± 1.81 in the Orenburg breed to 36.70 ± 6.09 in the Karachay breed. The ROHs included in class >0.8 Mb were the rarest in both breeds, and accounted for 0.58 ± 0.18 in the Orenburg breed and 2.55 ± 0.48 in the Karachay breed.

The mean length of ROHs per breed was the largest in class 0–0.5 Mb, corresponding to 78.43 ± 3.66 and 134.13 ± 18.79 in the Orenburg and Karachay breeds, respectively (Figure 5b). The mean length of ROHs included into class 0.5–1 Mb varied from 4.71 ± 0.57 in the Orenburg breed to 11.73 ± 2.08 in the Karachay breed. The lowest mean lengths of ROHs per breed were detected for class 1–2, as follows: 0.66 ± 0.20 and 0.13 ± 0.09 in the Orenburg and Karachay breeds, respectively.

### 3.3. Distribution of ROH Islands and Candidate Genes

**Figure 6:**
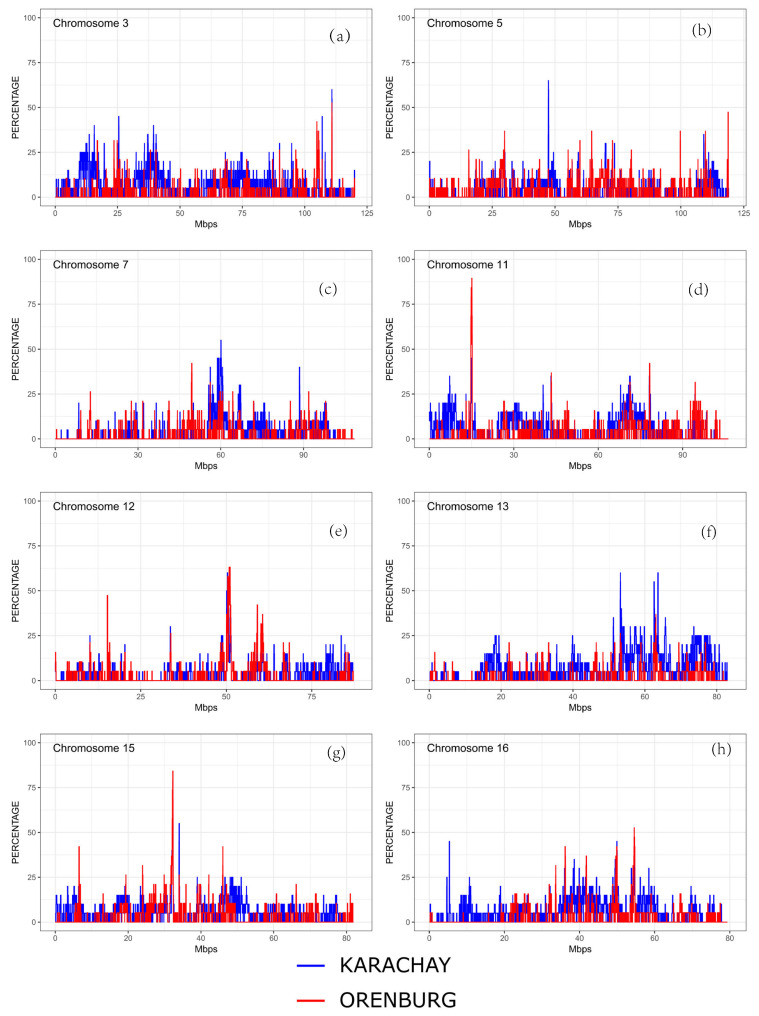
ROH islands identified in the Orenburg and Karachay breeds on the following chromosomes: (**a**) CHI3, (**b**) CHI5, (**c**) CHI7, (**d**) CHI11, (**e**) CHI12, (**f**) CHI13, (**g**) CHI15, and (**h**) CHI16.

We found ROH islands in the genome of the Orenburg breed on chromosomes CHI3, CHI11, CHI12, CHI15, and CHI16. In Karachay goats, ROH islands were identified on CHI3, CHI5, CHI7, CHI12, CHI13, and CHI15 (Figure 6).

For the most frequently occurring (in more than 50% of animals) islands of homozygosity, with an indication of the beginning and end of the regions, the number of SNPs in ROHs and the genes located in them are presented in Table 2.

The shared ROH islands were found on CHI12 (50,342,201–51,155,729 and 50,286,354–53,239,419 bp in the Orenburg and Karachay breeds, respectively) and on CHI15 (32,124,217–32,379,590 and 32,188,813–32,383,957 bp in the Orenburg and Karachay breeds, respectively). The former contained the genes encoding poly (ADP-ribose) polymerase family member 4 (*PARP4*) and M-phase phosphoprotein 8 (*MPHOSPH8*), whereas the latter included the genes for stromal interaction molecule 1 (*STIM1*) and ribonucleotide reductase catalytic subunit M1 (*RRM1*). The ROH segments on CHI11 spanning from 14,885,086 to 14,956,388 bp, 14,957,644 to 14,990,520 bp, 14,991,874 to 15,026,370 bp, and 15,064,670 to 15,155,143 bp and a ROH segment on CHI15 (32,269,497 to 32,363,003 bp) were common in ≥80% of the Orenburg breed.

## 4. Discussion

Traditional and autochthonous livestock breeds are an important reservoir of genetic diversity [51,52,53,54,55]. The Orenburg and Karachay breeds represent local goats that are well adapted for exploiting in a specific environment. The Orenburg breed is raised in steppes in continental climate conditions with strong winter winds and hot summers. The Karachay breed inhabits mountains and foothills in the North Caucasus region and may be successfully raised in the southern regions of Russia with higher summer temperatures. However, the genetic potential of these breeds has been consistently underestimated. Moreover, the Orenburg breed gene pool was threatened by the shutting down of gene pool farms [26,32].

In the present study, we examined the patterns of ROH distribution in these two Russian goat breeds based on WGS. Interestingly, Karachay goats exhibited larger genome coverage in ROHs compared to the Orenburg breed. This pattern is in accordance with our previous results obtained in a different sampling experiment of Karachay goats using DNA arrays [38]. According to Bertolini et al. [56], sometimes such a pattern may be observed in local breeds due to geographic isolation. In the case of the Orenburg goats examined in this study, we used samples from three geographically distant populations, which may affect the ROH coverage.

In general, the patterns of ROH distribution in Russian goats were compatible with the estimates in other goat breeds. For instance, the ROH length varied from 101.5 to 14,801.1 Kb in Mongolian cashmere goats [57]. In Swiss local breeds, the mean number of ROHs varied from 1174 to 1436, and the average length of ROHs ranged between 246 and 433 Kb [1]. In one study on Chinese goats [58], the average ROH length was 0.184 ± 0.102 Mb. The genomic coefficient (*F*_ROH_) values calculated in our study were in agreement with those obtained in Mongolian cashmere goats (0.026) [57], Swiss local goats (0.137–0.263) [1], Hainan black goats (0.107–0.186) [58], Latvian local goats (0.249) [4], and Ethiopian indigenous goats (0.016–0.261) [7]. Peng et al. [59] reported that the mean *F*_ROH_ values for African, European, and Bezoar goats were 0.073, 0.079 and 0.182, respectively.

We did not identify any long ROHs that reflect the recent inbreeding events [60]; moreover, our results are compatible with those obtained in Hu sheep, in the genome of which ROH segments with lengths from 300 Kb to 1 Mb were predominant [20]. Bian et al. [61] reported that ROHs were categorized into length classes of 0.5–1, 1–2, and 2–4 Mb in the Xiangxi white buffalo. A comparative genomic analysis of African zebu and taurine breeds revealed that the Holstein breed showed a significant accumulation of ROHs in all ROH classes, including 0.5–1 Mb, 1–2 Mb, and >2 Mb [22]. In Mongolian cashmere goats, the highest number of ROHs, representing 38.39% of the total ROH count, had the lengths of 0–0.5 Mb [57]. In Swiss local goats, the ROH segments longer than 1 Mb exceeded 50% of all detected ROHs [1]. ROHs with lengths of 0.1–0.2 Mb accounted for 72.41% of all ROH fragments, and ROHs with lengths over 1 Mb made up only 0.08% in Chinese goats [58]. Short ROHs (≤500 Kb) dominated in Latvian local goats [4]. Zhao et al. [20] assumed that short ROHs detected using WGS may point out the absence of inbreeding events in recent generations in livestock populations.

Based on the WGS data, we found ROH islands in both Russian breeds that harbored the potentially relevant candidate genes. The ROH island on CHI17 was common for both breeds and contained the *STIM1* and *RRM1* genes. *STIM1* was found to be under strong selection in many goat groups, including Swiss [1], Chinese [8,23,62,63], and Pakistani breeds [2,64]. The *STIM1* gene was related to body mass and weight in Teddy goats [2] and can be associated with horn/poll phenotypes in goats [63]. Sun et al. [23] suggested that *STIM1* was involved in genetic adaptations to local environmental conditions in Jintang Black goats. Wang et al. [8] identified a region harboring the STIM1 and *RRM1* genes potentially involved in reproduction in Jining gray goats. Furthermore, the region including *RRM1* and *STIM1* might be connected with gain in beef steers [65,66]. In addition, Sun et al. [23] reported that eight SNPs within the *STIM1* gene were fixed in several black goat breeds, while the Bezoars carry the mutant alleles. Also, Zheng et al. [67] found that the *STIM1*–*RRM1* haplotype in domestic goats was putatively introgressed from an ibex-like species and may be linked to neural function or behavior.

In both breeds, we found a ROH island on CHI12 that was wider in the Orenburg breed (~50.3–51.1 Mb) and contained the common *PARP4* and *MPHOSPH8* genes. This region overlapped, or was close to, the signatures of selection reported by Kim et al. [68], Bertolini et al. [56], Dadousis et al. [69], and Pegolo et al. [70] based on the identification of ROH hot spots. Kim et al. [68] suggested that this genomic region may be associated with adaptations to natural and climatic conditions. The ROH island identified in the Karachay breed, along with *PARP4* and *MPHOSPH8*, included the zinc finger MYM-type containing 2 (*ZMYM2*) gene. This gene was involved in DNA methylation patterning in the early embryonic development of mammals [71] and in the regulation of spermatogenesis and cell cycles in goats [72]. In addition, *ZMYM2* might be related to adaptive functions in Mediterranean sheep and goats [73]. It was also found in ROH islands in other Russian goat breeds [43], as well as in several sheep breeds [74].

A large group of genes, including those for gap junction protein β 2 (*GJB2*), gap junction protein β 6 (*GJB6*)***,*** crystallin lambda 1 (*CRYL1*), intraflagellar transport 88 (*IFT88*), interleukin 17 (*IL17D*), eukaryotic translation elongation factor 1 α lysine methyltransferase 1 (*EEF1AKMT1*), exp ortin 4 (*XPO4*), large tumor suppressor kinase 2 (*LATS2*), and Sin3A-associated protein 18 (*SAP18*), were identified in a ROH island in the Orenburg breed on CHI12. *GJB2* and *GJB6* regulate the formation of gap junction in the cochlea and play key roles in hearing [75]. Pegolo et al. [70] assumed that this region, found in ROH hot spots in diverse goat breeds, could have undergone selection even prior to the domestication of goats because enhanced hearing is beneficial to detect potential threats in natural habitats. Additionally, the *GJB2* gene, associated with body size and growth, may be subject to positive selection in the Boer [76] and Chinese goat breeds [77]. It was also found in the ROH islands in Chinese sheep breeds [20,78,79]. *GJB6* and *SAP18* may be involved in the regulation of reproductive performance in sheep by influencing ovarian and embryonic development, as well as spermatogenesis [19]. Quan et al. [80] proposed specific variants of the *GJB6* gene related to larger litter size as molecular markers for selective breeding.

The *CRYL1* gene is involved in the development of the nervous system and kidneys during embryogenesis in sheep [81] and in low mating behavior in Rasa Aragonesa rams [82]. Several studies have suggested a role of the *IFT88* gene in cashmere and mohair fineness in goats [83,84,85]. In addition, this gene plays an important role in spermatogenesis processes [86,87] and is under selection pressure in several Russian sheep breeds [88]. The *IL17D* gene is involved in the inflammatory response to respiratory infections [89]. Chen et al. [90] reported that the *LATS2* gene affected milk fat secretion and activation of lactogenesis in goats. In addition, this gene is involved in the regulation of reproduction functions in sheep [91] and cattle [92].

The genome regions under selection pressure were found in the Orenburg breed on CHI11 and CHI16. These contain the genes encoding Yip1 domain family member 4 (*YIPF4*), baculoviral IAP repeat containing 6 (*BIRC6*), tetratricopeptide repeat domain 27 (*TTC27*), and RAB GTPase activating protein 1-like (*RABGAP1L*). *YIPF4* might be associated with litter size in Dazu black goats [93]. In addition, the *YIP4* gene was proposed as a candidate gene that helps them potentially adapt to nutrient stress conditions [94]. The *TTC27* gene, associated with milk production traits, was identified to be under selection pressure in the Pakistani Teddy [2] and Dera-Din-Panah [64] goat breeds. *RABGAP1L* is involved in endocytosis and endosome maturation [95]. The *BIRC6* gene was identified in a ROH island in Chinese Cashmere goats [96] and was found to be under selection in Pakistani Teddy goats [2]. This gene was reported to play a role in the regulation of fertility traits and early embryonic development in different livestock species [97,98], including sheep [99]. In addition, the *BIRC6* gene was linked with body weight at 6 months in *Bos indicus* [100] and affected average daily gain in beef cattle [101].

Finally, a number of genes were identified in ROH islands in Karachay goats. These included signaling lymphocytic activation molecule family member 1 (*SLAMF1*), SIL1 nucleotide exchange factor (*SIL1*), pre-mRNA processing factor 6 (*PRPF6*), sterile α motif domain containing 10 (*SAMD10*), zinc finger protein 512B (*ZNF512B*), uridine–cytidine kinase 1 like 1 (*UCKL1*), tumor protein p53 inducible nuclear protein 2 (*TP53INP2*), nuclear receptor coactivator 6 (*NCOA6*), and γ-glutamyltransferase 7 (*GGT7*). *SLAMF1* is an immune-related gene in goats and other ruminant species [102,103]. The *SIL1* gene, related to immunity traits, was found in ROH islands in three indigenous Chinese cattle populations (Leiqiong, Lufeng, and Hainan) [104]. The *PRPF6* gene was associated with milk production traits in dairy goats [105]. *ZNF512B* may play a role in the regulation of feather morphogenesis in ducks [106]. The *TP53INP2* gene was involved in the positive regulation of differentiation in bovine adipocytes [107] and brown adipose tissue energy metabolism in humans [108]. Additionally, it was shown that the knockout of this gene increased skeletal muscle mass in mice [109,110]. *NCOA6* and *GGT7* were reported as candidates for fiber color in alpaca [111]. *NCOA6* was involved in the regulation of milk fat synthesis in dairy cattle and located within a quantitative trait locus for milk production traits [112,113].

## 5. Conclusions

In this study, we performed WGS in the Orenburg and Karachay goat breeds to find ROH islands under selective pressure and estimate an inbreeding level in their genomes. The observed *F*_ROH_ values in this study suggested low to moderate inbreeding in the studied breeds. The ROH islands were found on CHI3, CHI5, CHI7, CHI12, CHI13, and CHI15 in the Karachay goats; and on CHI3, CHI11, CHI12, CHI15, and CHI16 in the Orenburg breed. The shared ROH islands were found on CHI12 and on CHI15. Furthermore, we identified the candidate genes within the ROH islands. The results suggest that the majority of genes within one ROH island in the Karachay goats were associated with traits related to immunity, body weight, and milk production. Genes harboring in the ROH islands in the Orenburg goats were relevant to reproduction, increased litter size, growth, and development. In addition, a region on CHI11 that was common for 80% of the individuals and includes the *YIPF4*, *BIRC6*, and *TTC27* genes may be potentially considered as a target region for selection in the Orenburg breed. We thus identified candidate genes that reflect the essential biological specifics of the studied goat breeds. Our findings may be used for developing conservation strategies and for future genetic improvements in native Russian goats.

## Figures and Tables

**Table 1:** Runs of homozygosity (ROH) distribution values ^1^ in the Orenburg and Karachay breeds based on the WGS data analysis.

Breed	*n*	ROH Length			ROH Number			*F*_ROH_		
		Mean ± SE	Min	Max	Mean ± SE	Min	Max	Mean ± SE	Min	Max
Orenburg	19	78.43 ± 3.66	47.57	102.96	438.79 ± 16.83	283	540	0.032 ± 0.001	0.02	0.04
Karachay	20	134.13 ± 18.79	30.38	404.40	695.25 ± 94.20	189	2075	0.054 ± 0.008	0.01	0.16

^1^ SE, standard error; Min, minimum; Max, maximum.

**Table 2:** Genes located in the ROH islands (common in ≥50% in each breed) in the Orenburg and Karachay breeds based on the WGS analysis.

Breed	CHI	Start, bp	End, bp	No. of SNPs	Gene
Orenburg	3	110,894,406	110,897,532	9	
	11	14,760,399	15,261,320	1358	*YIPF4*, *BIRC6*, *TTC27*
	12	50,342,201	50,432,910	168	***PARP4*, *MPHOSPH8***
	12	50,530,724	50,533,436	10	
	12	50,671,450	50,805,766	459	*GJB2*, *GJB6*, *CRYL1*
	12	50,809,376	50,947,946	425	*IFT88*, *IL17D*, *EEF1AKMT1*
	12	50,948,756	50,965,731	50	
	12	50,968,611	51,033,025	133	*XPO4*, *LATS2*
	12	51,035,319	51,155,729	386	*SAP18*
	15	32,124,217	32,379,590	394	***STIM1*, *RRM1***
	16	54,524,824	54,531,631	20	*RABGAP1L*
Karachay	3	110,862,077	110,964,407	226	*SLAMF1*
	5	47,322,478	47,471,119	626	
	7	60,029,670	60,088,713	184	*SIL1*
	7	60,090,256	60,138,223	128	
	12	50,286,354	50,432,910	292	***PARP4*, *MPHOSPH8***
	12	50,530,724	50,537,889	24	
	12	50,543,508	50,573,061	41	*ZMYM2*
	13	53,173,841	53,239,419	116	*PRPF6*, *SAMD10*, *ZNF512B*, *UCKL1*
	13	62,528,703	62,629,819	327	
	13	63,614,834	63,737,342	398	*TP53INP2*, *NCOA6*, *GGT7*
	15	32,188,813	32,383,957	400	***STIM1*, *RRM1***

Genes in bold were found in both breeds.

## Data Availability

The WGS data for Orenburg and Karachay goats used in this study are available upon request from the corresponding author.
